# Management of First-Time Anterior Shoulder Dislocation—A Systematic Review and Meta-analysis: Arthroscopy Association of Canada Position Statement

**DOI:** 10.1177/23259671251316893

**Published:** 2025-02-06

**Authors:** Hassaan Abdel Khalik, Danielle Dagher, Darius Luke Lameire, Eva Gusnowski, Michaela Kolpka, Marie-Eve LeBel, Bogdan A. Matache, R. Kyle Martin, Mark Sommerfeldt, Ivan Wong, Jarret Woodmass, Moin Khan

**Affiliations:** * McMaster University, Hamilton, Ontario, Canada; † University of Toronto, Toronto, Ontario Canada; ‡ Saint John Orthopaedics, Saint John, New Brunswick, Canada; § Banff Sport Medicine, Canmore, Alberta, Canada; ‖ Western University, London, Ontario, Canada; ¶ Laval University, Quebec City Quebec, Canada; # University of Minnesota, Minneapolis, Minnesota, USA; ** University of Alberta, Edmonton, Alberta, Canada; †† Nova Scotia Health Authority, Halifax, Nova Scotia, Canada; ‡‡ University of Manitoba, Winnipeg, Manitoba, Canada; Investigation performed at McMaster University, Hamilton, Ontario, Canada

**Keywords:** anterior shoulder instability, anterior shoulder dislocation, arthroscopic Bankart repair, shoulder stabilization surgery

## Abstract

**Background:**

While surgical stabilization is typically recommended for patients with recurrent shoulder instability, the management of first-time shoulder dislocation (FTSD) presents a unique challenge for health care providers.

**Purpose:**

To assess the efficacy of arthroscopic Bankart repair (ABR) compared with nonoperative management for FTSDs.

**Study Design:**

Review.

**Methods:**

MEDLINE, EMBASE, and CENTRAL were searched from inception to December 26, 2023, for comparative studies assessing ABR versus nonoperative management of FTSDs. Outcomes of interest included rates of shoulder redislocation, cumulative shoulder instability (redislocation, subluxation, and/or subjective instability), subsequent shoulder stabilization surgery, return-to-sport rates, and patient-reported outcomes (Western Ontario Shoulder Instability [WOSI] score and Rowe score). Meta-analyses were performed on outcomes reported across a minimum of 3 comparative studies.

**Results:**

Eleven comparative studies with 694 patients (695 shoulders) were included in the final analysis. Patient demographics were comparable across arthroscopic stabilization (367 shoulders) and nonoperative management (328 shoulders) groups with a mean age of 21.6 ± 2.5 years across all studies, and 13.7% ± 13.6% of patients being female. The mean follow-up across all studies was 54.2 ± 28.5 months, with a mean loss to follow-up of 8.1% ± 10.3%. Meta-analyses demonstrated a reduction in the odds of cumulative instability in favor of the ABR group (odds ratio [OR], 0.04 [95% CI, 0.02 to 0.08]; *P* < .01), as well as reductions in the odds of shoulder redislocation (OR, 0.06 [95% CI, 0.02 to 0.17]; *P* < .01) and subsequent stabilization surgery (OR, 0.07 [95% CI, 0.03 to 0.14]; *P* < .01) in favor of ABR. Compared with the nonoperative group, patients in the ABR group were 3.87 times more likely to return to sport at the preoperative or higher level (OR, 3.87 [95% CI, 1.57 to 9.52]; *P* < .01). No differences were found across postoperative WOSI scores (mean difference, 8.08 [95% CI, –1.54 to 17.69]; *P* = .10). Subgroup analyses demonstrated similar outcomes between randomized controlled trials and observational studies.

**Conclusion:**

Early ABR of first-time anterior shoulder dislocations consistently demonstrated decreased subsequent rates of cumulative instability events, shoulder redislocations, and revision surgeries relative to nonoperative management.

The shoulder is the most commonly dislocated joint in the body, with shoulder dislocations accounting for half of all major joint dislocations.^2^ Anterior shoulder dislocations are the most common, representing approximately 97% of all shoulder dislocations.^2^ Their incidence rate is higher in young and active individuals, and the condition primarily affects male patients.^32,38,58^ Anterior shoulder dislocations are often complicated by recurrent instability and redislocations.^32,43,46^ Biomechanical data has shown that after a first dislocation, injury to the joint alters the normal biomechanics and increases the risk of recurrent instability. This, in turn, can lead to further joint damage that is amplified with each subsequent dislocation,^37,57^ highlighting the need for timely and effective management. While surgical stabilization is typically recommended for patients with recurrent instability (≥2 dislocations), the optimal management of first-time shoulder dislocation (FTSD) remains controversial and presents a unique challenge for health care providers.

Two initial treatment options exist for patients with an FTSD: nonoperative management and surgical stabilization. Currently, the vast majority of FTSDs are initially treated conservatively with a brief period of immobilization followed by a functional rehabilitation protocol.^25^ Proponents of this approach argue that around 50% of FTSDs can be effectively managed nonoperatively and that patients at higher risk for recurrent instability (younger, male, partaking in contact sports, high degree of bone loss) can be identified appropriately and offered surgical management, while the remainder can be treated nonoperatively.^26,48^ Advocates for primary surgical stabilization argue that rates of redislocation after primary nonoperative management are high enough to warrant immediate operative repair of the dislocated joint, particularly for young and active patients.^19^ Additionally, delay in effective treatment causes further damage to the joint, increasing the surgical complexity and associated risk of subsequent treatment failure, as well as potentially increasing the risk of future shoulder arthropathy.^14,20,34,37,57^ Arthroscopic Bankart repair (ABR) is a soft tissue stabilization procedure commonly used to treat anterior shoulder instability, particularly in the setting of minimal bone loss.^24,39^ Arthroscopic techniques are minimally invasive and have evolved to demonstrate comparable postoperative outcomes when compared with open techniques.^22,23^

The aim of this review was to evaluate the efficacy of ABR compared with nonoperative management for FTSDs. This position statement provides a synthesis of available evidence in the form of a systematic review and meta-analysis and is intended to help guide decision making for this challenging clinical scenario.

## Methods

This systematic review was performed according to the guidelines set by the Cochrane handbook^18^ and the PRISMA (Preferred Reporting Items for Systematic Reviews and Meta-Analyses).^36^

### Comprehensive Search Strategy

MEDLINE, EMBASE, and CENTRAL (Cochrane Central Register of Controlled Trials) were electronically searched from database inception through December 26, 2023, by 2 reviewers (H.A.K. and D.D.) for literature related to arthroscopic stabilization surgery for anterior FTSDs. Search terms utilized included “first-time shoulder dislocation,”“arthroscopic,” and “stabilization surgery” (Appendix Table A1).

The inclusion criteria for this review were (1) ABR surgery, (2) stabilization surgery occurring after a first-time anterior shoulder dislocation, (3) comparative studies, (4) clinical and/or functional outcomes reported, (5) both skeletally mature and skeletally immature patient populations, (6) human studies, and (7) English-language publication. Excluded from this review were (1) biomechanical studies and (2) review articles. In situations where multiple studies included the same population, the most recent study was selected for inclusion, although preference was given to comparative data.

### Study Screening

Two authors (H.A.K. and D.D.) independently screened studies at both the title/abstract and the full-text stages of the review. To prevent premature exclusion, disagreements at the title/abstract stage were advanced to the full-text stage. Conflicts at the full-text stage were resolved by the senior author (M.K.). The kappa (κ) score was calculated after each step of the screening process to determine the level of agreement between reviewers. The categorization of κ values was defined a priori according to Landis and Koch^30^ as follows: 1.00 > κ≥ 0.81 indicated almost perfect agreement, 0.80 > κ≥ 0.61 indicated substantial agreement, 0.60 > κ≥ 0.41 indicated moderate agreement, 0.40 > κ≥ 0.21 indicated fair agreement, 0.20 > κ≥ 0.00 indicated slight agreement, and κ = 0 indicated no agreement.

### Assessment of Study Quality

The risk of bias and quality assessment of included studies was independently assessed by 2 authors (H.A.K. and D.D.). The Cochrane risk-of-bias tool was used to assess risk of bias for randomized controlled trials (RCTs),^17^ and the methodological index for non-randomized studies (MINORS) was used for quality assessment for the non-RCTs.^45^ The Cochrane risk-of-bias tool consists of 6 domains including selection bias, performance bias, detection bias, attrition bias, reporting bias, and other bias.^17^ Reviewers rate the bias in each domain as either high risk, unclear risk, or low risk. The MINORS questionnaire consists of 8 items for noncomparative studies, and 4 additional items for comparative studies. Each item is given a score of 2 if reported and adequate, 1 if reported but inadequate, and zero if not reported. Noncomparative studies can have a maximum score of 16, and comparative studies can have a maximum score of 24.

### Data Abstraction From the Included Studies

Data abstraction was performed by 3 reviewers (H.A.K., D.D., and D.L.L.). Each reviewer independently abstracted the data from one-half of the studies while the other reviewed the accuracy of the abstracted data. The data were abstracted into a Google Sheets spreadsheet designed a priori. The following data were abstracted from the included studies: study characteristics (authors, study design, publication year, etc), number of patients, patient demographics (age, sex, etc.), follow-up length, postoperative complication rates, recurrent shoulder redislocation rates, cumulative instability rates, rates of subsequent stabilization surgery, and return to sport/work rates, as well as subjective and objective outcome measures. The level of evidence for the studies was determined based on the American Academy of Orthopaedic Surgeons evidence-based practice committee guidelines.^54^

Outcomes of interest included rates of shoulder redislocation, cumulative instability, reoperation after arthroscopic shoulder stabilization, rate of return to sport, index surgery complication rates, and patient-reported outcomes. Cumulative instability rates were defined as being inclusive of redislocation, subjective feelings of instability, and/or subluxation events.^6,24,50^ Shoulder redislocation rates were presented as an additional, separate value only if they were explicitly reported as such by included studies. Patient-reported outcomes included the Western Ontario Shoulder Instability Index (WOSI) score and the Rowe score.^27,42^

### Statistical Analysis

Descriptive statistics were calculated using R (RStudio). These included weighted means with standard deviations as well as 95% CIs. Depending on the outcome, either the number of patients or the number of shoulders in each study was used as the frequency weight.

A pairwise meta-analysis was performed on all outcomes reported across ≥3 studies using a DerSimonian and Laird random-effects model.^12^ Odds ratios (ORs) were calculated for dichotomous variables and mean difference was calculated for continuous variables, along with their respective 95% CIs to confirm the effect size estimation. For studies reporting median and interquartile range, the mean and standard deviation was estimated using a previously outlined method by Wan et al.^52^ Missing standard deviations were calculated as per the methodology of the Cochrane Handbook for Systematic Reviews of Interventions Version 6.1.^18^ The meta-analysis was performed on DataParty. Meta-analyses were stratified by study design (RCTs vs observational studies). Heterogeneity was quantified using the chi-square test for heterogeneity and the *I*^2^ statistic. We considered *I*^2^ values of <25% to indicate low heterogeneity and >75% to indicate considerable heterogeneity. Subgroup analyses were also planned for studies to evaluate the effect of study design. Outcomes not amenable to meta-analysis were presented in a narrative manner.

## Results

### Study Characteristics and Quality

**Figure 1.**
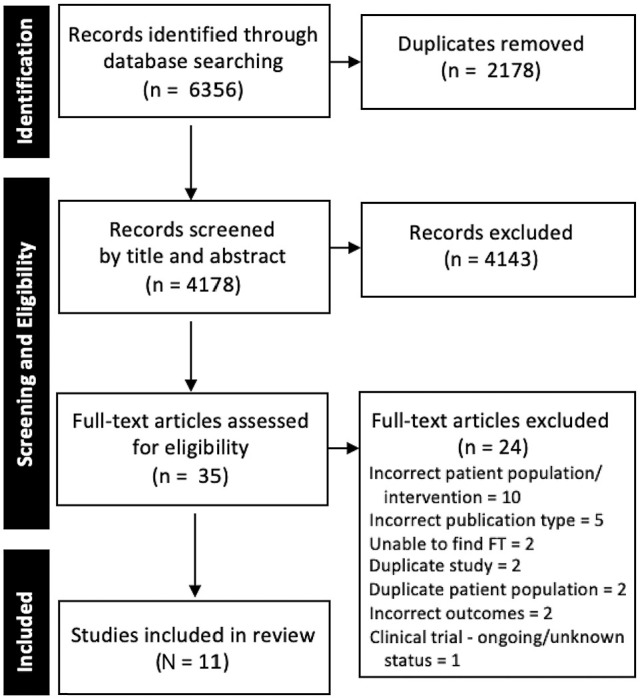
PRISMA (Preferred Reporting Items for Systematic Reviews and Meta-Analyses) diagram of the study inclusion process. FT, full text.

**Figure 2.**
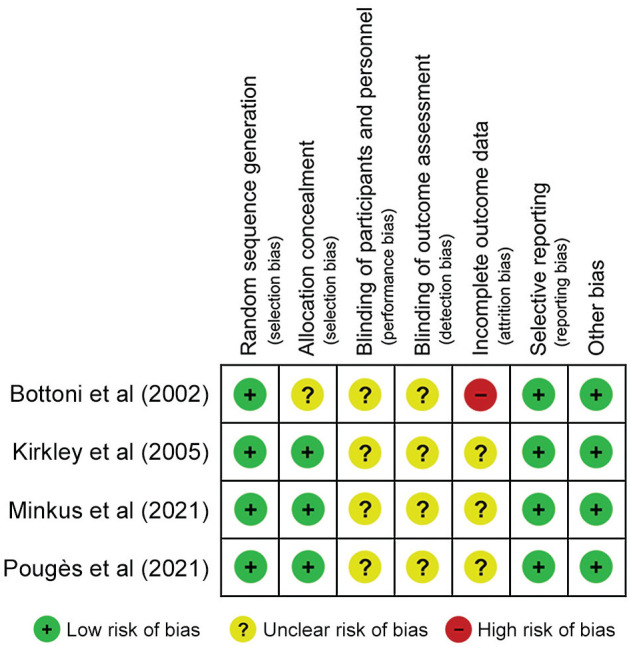
Risk-of-bias assessment for the included randomized controlled trials.^7,28,35,41^

The search identified 6356 studies, with 4178 remaining after exclusion of duplicates. After screening, 11 studies^¶¶^ were included in the final analysis (Figure 1). Both the title/abstract and full-text stages had almost perfect interobserver agreement (κ = 0.81 [95% CI, 0.70-0.92] and κ = 0.88 [95% CI, 0.71-1.00], respectively). The 4 included RCTs^7,28,35,41^ were all rated as having an unclear risk of bias (Figure 2). All 4 RCTs commonly lacked blinding of participants and personnel and blinding of outcome assessment, and they had a significant loss to follow-up potentially resulting in attrition bias. The mean MINORS score for the included non-RCTs was 17.4 (range, 14-21) (Table 1). The characteristics of the included studies are summarized in Table 1.

**Table 1:** Overview of Included Studies (N = 11)^*a*^

Study, Lead Author (year)	Study Design, LOE	Intervention Groups	Patients/ Shoulders, n	Age, y^*b*^	Female, %	Follow-up, mo^*b*^	Loss to Follow-up, %	Time From Dislocatito Arthroscopic Stabilization	MINORS Score
Arciero (1994)^5^	Prospective cohort; 3	(1) Nonop (2) ABR	(1) 15/15 (2) 21/21	(1) 19.5 (18-21) (2) 20.5 (18-24)	(1) 0 (2) 0	(1) 23 (15-39) (2) 32 (15-45)	(1) 0 (2) 0	(1) NA (2) Mean, 5.5 d after dislocation	20
Bottoni (2002)^7^	RCT; 1	(1) Nonop (2) ABR	(1) 14/14 (2) 10/10	(1) 23 (19-26) (2) 21.6 (19-26)	(1) 0 (2) 0	(1) 37 (16-56) (2) 35 (17-56)	(1) 14.3 (2) 10	(1) NA (2) ≤10 d after injury	NA
De Carli (2019)^10^	Prospective cohort; 2	(1) Nonop (2) ABR	(1) 96/96 (2) 64/64	(1) 20.8 ± 2.9 (2) 22.8 ± 3.2	(1) 34.4 (2) 9.4	(1) 103.6 (24-149) (2) 82.3 (24-134)	(1) 27.1 (2) 6.3	(1) NA (2) Decision for surgery ≤1 wk	20
DeBerardino (2001)^11^	Prospective cohort; 2	(1) Nonop (2) ABR	(1) 6/6 (2) 48/49	(1) NR (2) 20 (17-23)	(1) NR (2) 6.3	(1) NR (2) 37 (24-60)	(1) 0 (2) 0	(1) NA (2) ≤10 d after injury	15
Gigis (2014)^15^	Prospective cohort; 2	(1) Nonop (2) ABR	(1) 27/27 (2) 38/38	(1) 16.6 (2) 16.6	(1) 37 (2) 36.8	(1) 36 (2) 36	(1) 0 (2) 0	(1) NA (2) ≤21 d after injury	14
Kirkley (2005)^28^	RCT; 2	(1) Nonop (2) ABR	(1) 15/15 (2) 16/16	(1) 22.7 (2) 23.3	(1) 6.7 (2) 6.3	79^*c*^ (51-102)	(1) 28.6 (2) 15.8	(1) NA (2) ≤4 wk after dislocation	NA
Larrain (2001)^31^	Prospective cohort; 2	(1) Nonop (2) ABR	(1) 18/18 (2) 28/28	21^*c*^ (17-27)	NR	67.4^*c*^ (28-120)	0	(1) NA (2) NR	16
Minkus (2021)^35^	RCT; 1	(1) Nonop (2) ABR	(1) 60/60 (2) 52/52	(1) 26.7 ± 5.8 (2) 25.7 ± 6.2	(1) 8.3 (2) 7.7	24	(1) 21.7 (2) 15.4	(1) NA (2) ≤3 wk after injury	NA
Pougès (2021)^41^	RCT; 1	(1) Nonop (2) ABR	(1) 20/20 (2) 20/20	(1) 21.3 (20-22.5) (2) 21.5 (20.5-22.5)	(1) 10 (2) 25	(1) 24 (2) 24	(1) 5 (2) 5	(1) NA (2) ≤15 d after injury	NA
Shih (2011)^44^	Prospective cohort; 2	(1) Nonop (2) ABR	(1) 25/25 (2) 39/39	(1) 22.9 (17-29) (2) 21.5 (18-29)	(1) 0 (2) 0	(1) 70.4 (60-84) (2) 72.6 (62-88)	(1) 0 (2) 0	(1) NA (2) Mean, 5 d (range, 1-12 d)	21
Yanmis (2003)^55^	Prospective cohort; 2	(1) Nonop (2) ABR	(1) 32/32 (2) 30/30	(1) 22.1 (19-32) (2) 21.2 (18-26)	(1) 12.5 (2) 0	(1) 40 (18-63) (2) 33 (10-60)	(1) 0 (2) 0	(1) NA (2) Mean, 10.5 d (range, 5-23 d)	16

a ABR, arthroscopic Bankart repair; LOE, level of evidence; MINORS, methodological index for non-randomized studies; NA, not applicable; Nonop, nonoperative; NR, not reported; RCT, randomized controlled trial.

b Data are presented as mean, mean (range), or mean ± SD, unless otherwise indicated.

c Median.

A total of 694 patients (695 shoulder dislocations) were included in this review (Table 1). The mean age of patients across all studies was 21.8 ± 2.6 years, with 13.7% ± 13.6% of participants being female. Comparable patient and study demographics were seen across both ABR and nonoperative groups (Table 2). The mean follow-up across all studies was 54.2 ± 28.5 months, with a mean loss to follow-up of 8.1% ± 10.3%. The mean time to surgery for participants in the ABR group was 7.0 ± 2.5 days, reported in 3 studies.^5,44,55^ The remaining studies reported threshold time frames within which surgery took place, ranging from within 10 days to 4 weeks from initial injury (Table 1).

**Table 2:** Summary of Characteristics of Patients in the Included Studies Overall and by Intervention Group^*a*^

Characteristic	All	Arthroscopic Bankart Repair	Nonoperative Management
Intervention arms, n	22	11	11
Initial shoulder dislocations, n	695	367	328
Age, y	21.8 ± 2.6 (20.7-22.9)	21.6 ± 2.5 (20.1-23.2)	22.1 ± 2.8 (20.2-23.9)
Female, %	13.7 ± 13.6 (7.2-20.1)	8.8 ± 10.9 (1.7-16)	19.0 ± 14.1 (9.2-28.8)
Follow-up, mo	54.2 ± 28.5 (42.0-66.4)	46.5 ± 22.6 (31.8-61.3)	57.9 ± 34.5 (34.0-81.8)
Loss to follow-up, %	8.1 ± 10.3 (3.8-12.3)	3.8 ± 5.5 (0.6-7.0)	12.8 ± 12.1 (5.6-20.0)

a Data are presented as mean ± SD (95% CI) unless otherwise indicated.

### Outcomes

#### Subsequent Instability, Redislocation, and Surgery

**Figure A1.**
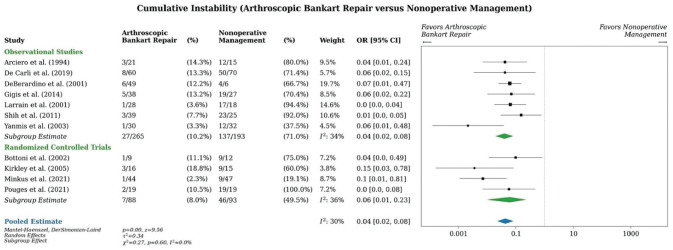
Results of meta-analysis on cumulative instability.

Cumulative subsequent instability rates were 9.6% ± 4.8% and 64.0% ± 25.7% across ABR and nonoperative groups, respectively (Table 3). All 11 included studies^¶¶^ were included in the meta-analysis, which demonstrated that the odds of cumulative subsequent instability were decreased by 96% when FTSDs were treated with ABR compared with nonoperative management (OR, 0.04 [95% CI, 0.02-0.08]; *P* < .01) (Appendix Figure A1). This trend was consistent across both observational studies and RCTs.

**Table 3:** Summary of Primary Outcomes

Outcome	Arthroscopic Bankart Repair		Nonoperative Management		All	
	No. of Studies	Mean ± SD (95% CI)	No. of Studies	Mean ± SD (95% CI)	No. of Studies	Mean ± SD (95% CI)
Shoulder redislocation	9	6.2 ± 5.3 (2.8-9.7)	9	50.6 ± 30.2 (30.9-70.3)	18	26.0 ± 30.2 (12.1-40)
Cumulative subsequent instability	11	9.6 ± 4.8 (6.8-12.5)	11	64.0 ± 25.7 (48.8-79.2)	22	34.0 ± 32.2 (20.5-47.4)
Subsequent stabilization surgery	8	6.1 ± 3.7 (3.5-8.7)	7	39.2 ± 20.9 (23.7-54.6)	15	19.7 ± 21.2 (8.9-30.4)

**Figure A2.**
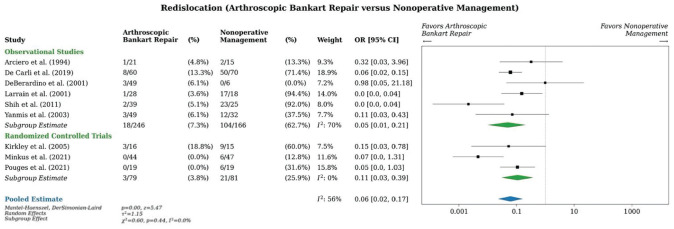
Results of meta-analysis on redislocation rate.

Shoulder redislocation rates were 6.2% ± 5.3% and 50.6% ± 30.2% across ABR and nonoperative groups, respectively. Overall, 9 studies^##^ were eligible for meta-analysis, which demonstrated that the odds of redislocation were 94% lower in the ABR group compared with the nonoperative group (OR, 0.06 [95% CI, 0.02-0.17]; *P* < .01) (Appendix Figure A2).

**Figure A3.**
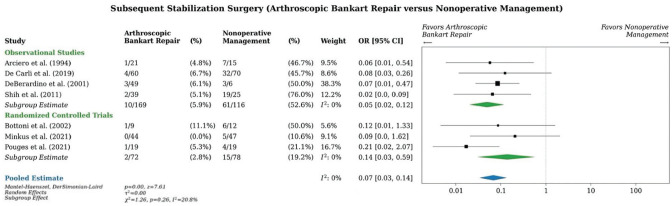
Results of meta-analysis on subsequent stabilization surgery.

Rates of subsequent stabilization surgery were 6.1% ± 3.7% and 39.2% ± 20.9% across ABR and nonoperative groups, respectively (Table 3). Meta-analysis performed on 7 eligible studies^5,7,10,11,35,41,44^ demonstrated that the odds of undergoing subsequent stabilization surgery were 93% smaller in FTSDs managed with ABR compared with nonoperative management (OR, 0.07 [95% CI, 0.03-0.14]; *P* < .01) (Appendix Figure A3).

The rate of postoperative complications after ABR was 1.8% ± 4.3% across 6 studies.^5,10,11,31,35,41^

#### Return to Sport

**Figure A4.**
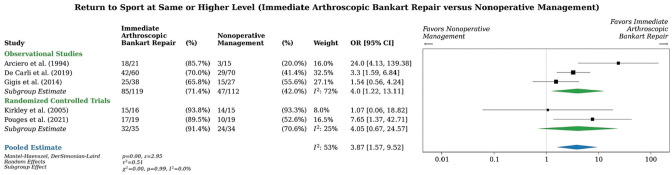
Results of meta-analysis on return to sport at the same or higher level.

Return to sport at preoperative levels or higher was 79.2 ± 10.7 and 48.5 ± 18.1 for ABR and nonoperative groups, respectively (Table 4). Meta-analysis performed on 5 studies^5,10,15,28,41^ demonstrated that patients in the ABR group were 3.87 times more likely to return to sport at preoperative levels or higher when compared with nonoperative management (OR, 3.87 [95% CI, 1.57-9.52]; *P* < .01) (Appendix Figure A4). Two studies^10,41^ assessed return-to-sport rates at any level, which precluded a meta-analysis, although rates were comparable across ABR and nonoperative management (93.7% ± 0.7% and 84.2% ± 8.4%, respectively).

**Table 4:** Return-to-Sport Outcomes^*a*^

Study	n^*b*^	Sporting Activity Level, n (%)	Return to Sport, %		
			At Same/Higher Level	At Any Level	Outcomes
Arthroscopic Bankart Repair					
Arciero (1994)^5^	21	Varsity/club: 8 (38) Intramural: 6 (29) Required physical activity: 7 (33)	85.70		NR
De Carli (2019)^10^	60	Professional: 12 (20) Amateur: 16 (27) Recreational: 32 (53)	70.00	93.30	• RTS at any level: *P* = .35 • RTS at same/higher level: *P* = .001
DeBerardino (2001)^11^	49	Military cadets: 49 (100)	89.60		NR
Gigis (2014)^15^	38	NR	65.80		No significance
Kirkley (2005)^28^	16	NR	93.80		NR
Pougès (2021)^41^	20	None: 5 (25) Recreational: 9 (45) Competition: 6 (30)	89.00	95.00	RTS at same/higher level: *P* = .12
Overall mean ± SD (95% CI)			79.2 ± 10.7 (70.6-87.8)	93.7 ± 0.7 (92.7-94.7)	
Nonoperative Management					
Arciero (1994)^5^	15	Varsity/club: 10 (67) Intramural: 2 (13) Required physical activity: 3 (20)	20.00		NR
De Carli (2019)^10^	70	Professional: 12 (17) Amateur: 32 (46) Recreational: 26 (37)	41.40	88.60	• RTS at any level: *P* = .35 • RTS at same/higher level: *P* = .001
Gigis (2014)^15^	27	NR	55.60		No significance
Kirkley (2005)^28^	15	NR	93.30		NR
Pougès (2021)^41^	20	None: 2 (10) Recreational: 14 (70) Competition: 4 (20)	53.00	68.00	RTS at same/higher level: *P* = .12
Overall mean ± SD (95% CI)			48.5 ± 18.1 (32.6-64.4)	84.2 ± 8.4 (72.5-95.9)	

a NR, not reported; RTS, return to sport.

b Number of shoulders at final follow-up.

#### Patient-Reported Outcomes

Mean WOSI scores were 91.5 ± 2.9 and 82.2 ± 6.5 for ABR and nonoperative groups, respectively, with a higher score representing better outcomes (Table 5). Meta-analysis across 5 eligible studies^10,28,35,41,44^ demonstrated a pooled mean difference of 8.08 points in favor of the ABR group (95% CI, –1.54 to 17.69; *P* = .10).

**Table 5:** Patient-Reported Outcome Scores^*a*^

Study	Arthroscopic Bankart Repair		Nonoperative Management		*P*
	n^*b*^	Score	n^*b*^	Score	
WOSI					
De Carli (2019)^10^	60	94.5 ± 4.0	70	76.5 ± 5.2	<.001
Kirkley (2005)^28^	16	86	15	74.8	.17
Minkus (2021)^35^	44	92.7 ± 8.1	47	91.5 ± 7.9	NS
Pougès (2021)^41^	19	88.5	19	82.3	.04
Shih (2011)^44^	39	89.1	25	84.9	.18
Overall mean ± SD (95% CI)		91.5 ± 2.9 (88.9-94.0)		82.2 ± 6.5 (76.5-87.8)	
Rowe					
Arciero (1994)^5^	21	Excellent: 16 (76.2) Good: 2 (9.5) Poor: 3 (14.3)	15	NR	NA
De Carli (2019)^10^	60	94.2 ± 5.3	70	75.3 ± 5.5	<.001
DeBerardino (2001)^11^	49	92 (30-100)	6	NR	NA
Larrain (2001)^31^	28	Excellent: 25 (89.3) Good: 2 (7.1) Poor: 1 (3.6)	18	Excellent: 1 (5.6) Poor: 17 (94.4)	NR
Minkus (2021)^35^	44	88.5 ± 11.2	47	89.1 ± 7.1	NS
Overall mean ± SD (95% CI)		91.9 ± 2.3 (89.2-94.5)		80.8 ± 6.8 (71.5-90.2)	

a Data presented as mean ± standard deviation (range) or n (%), unless otherwise indicated; NA, not applicable; NR, not reported; NS, not significant; WOSI, Western Ontario Shoulder Instability Index.

b Number of patients at final follow-up.

Mean postoperative Rowe scores were 91.9 ± 2.3 and 80.8 ± 6.8 for ABR and nonoperative groups, respectively (Table 5). Only 2 studies reported postoperative mean values, precluding meta-analysis: DeCarli et al^10^ reported results largely in favor of ABR compared with nonoperative management (94.2 ± 5.3 vs 75.3 ± 5.5, respectively; *P* < .001), while Minkus et al^35^ reported results in favor of nonoperative management compared with ABR (89.1 vs 88.5, respectively), although this difference was negligible.

## Discussion

In this review, we aimed to evaluate the effects of ABR compared with nonoperative management in patients with an anterior FTSD. We included 11 studies with 694 patients (695 shoulders). The main findings were that ABR decreased the odds of subsequent instability, redislocation, and subsequent stabilization surgery compared with nonoperative management. Patients who underwent ABR were also more likely to return to sport at preoperative levels or higher. WOSI scores favored ABR, with the observed mean difference surpassing the minimally clinically important difference for WOSI scores reported in the literature.^40^

### Relation to Previous Literature

The results of this review are consistent with previous literature. A review by Hurley et al^24^ found concordant results, reporting that ABR resulted in a lower recurrence rate and higher rate of return to sport when compared to non-operative management. Hurley et al^24^ also noted that patients who underwent ABR experienced low complication rates, similar to the results of the current review (1.6% and 1.8%, respectively). A more recent meta-analysis by Hu et al^21^ further solidifies these findings, concluding that ABR showed superiority over conservative management with respect to recurrence, return to play, and subsequent instability surgery in young, active patients presenting with an FTSD. Additionally, Kraeutler et al^29^ reported that operative management for anterior FTSDs can both reduce the recurrence rate and provide improved quality of life postoperatively, particularly in younger patients. A review by Longo et al^33^ found similar results in pediatric populations (aged ≤18 years), reporting lower recurrence rates in patients who underwent surgical treatment.

Our findings contribute to this body of evidence by further demonstrating the benefits of ABR compared with nonoperative approaches. Specifically, our review emphasized the consistent improvement in recurrence rates, return to sport, and overall shoulder stability. While prior systematic reviews and meta-analyses do exist on this topic, their strength and the generalizability of their conclusions may be questioned due to the methodology utilized. For example, a prior review by Adam et al^3^ included comparative studies including studies comparing ABR after a single dislocation compared with either nonoperative management or Bankart repair after recurrent dislocation, 2 inherently different populations. Another meta-analysis by Alkhatib et al^4^ only included 6 RCTs for analysis and did not stratify between cumulative instability and confirmed redislocation events. Similarly, a study by Hurley et al^24^ across prospective studies did not delineate between cumulative instability and redislocation events. Ultimately, the agreement of this meta-analysis with existing literature along with the granularity of provided data underscores the reliability of ABR as an effective intervention and provides a strong foundation for future research to explore optimal surgical techniques and rehabilitation protocols.

### Consensus Statements and Risk Factors

Various consensus statements have sought to standardize the diagnosis and treatment of FTSDs, especially considering the heterogeneity of presenting patients. A notable consensus statement on the management of FTSDs utilizing the Delphi approach was by the Neer Circle of American Shoulder and Elbow Surgeons, wherein they presented recommendations for all patients as well as stratified by the following categories: (1) active-duty military population, (2) nonathlete population, (3) noncontact athlete population, and (4) contact athlete population.^47^ Patients <14 or >30 years of age had a decreased likelihood of being operatively managed irrespective of category. Strong predictors of surgery across all patient categories were meaningful bone loss as well as a positive apprehension test. Interestingly, the inclusion of bone loss in the final survey was debated by committee members as not being clinically relevant, though prior studies have demonstrated the rate of bone loss after an FTSD to be as high as 41%.^16^ Nonetheless, the degree of bone loss may not be clinically relevant, with Dickens et al^13^ demonstrating that the mean amount of glenoid bone loss after a single anterior shoulder dislocation was only 6.5%. Therefore, it is up to clinicians to weigh the benefits and risks of proceeding with computed tomography (CT) imaging with the associated radiation exposure in often young patients to accurately assess bone loss.

Another notable Delphi study by the Anterior Shoulder Instability International Consensus Group asked their participants about the ordering of advanced imaging in the setting of anterior shoulder instability.^25^ Strong consensus (90%) existed regarding ordering advanced imaging (CT scan or magnetic resonance imaging [MRI]) in patients deemed to have a high risk of recurrence, and moderate consensus (84%) existed for ordering a CT scan if there was concern for bone loss.^25^ While this study did not explicitly assess patient factors associated with high risk of recurrence, robust evidence to-date suggests male sex, younger age, and ligamentous laxity are associated with recurrent instability after a single dislocation event.^38^ Further, while this consensus statement provided recommendations for the management of anterior shoulder instability, their recommendations were not specifically directed at the patient experiencing an FTSD.

Ultimately, while the current review demonstrated strong evidence in favor of surgical management of FTSDs, there are several considerations that cannot be captured in RCTs, including but not limited to a patient’s functional objectives, whether the patient is in-season with strict return to sport timelines, and associated bone loss. Therefore, it is recommended by the Arthroscopy Association of Canada (AAC) to consider surgical management in patients with FTSD in the presence of certain risk factors, including male sex, young age at first dislocation, ligamentous laxity, and participation in contact sport, through a shared decision-making process with the patient.^38,53^

### Methodological Limitations

Consensus statements and expert surveys are restricted by the methodological limitations of the available literature, including statistically fragile results as well as the limited generalizability of findings. The development and execution of high-quality trials to assess operative versus nonoperative management of FTSDs are challenging due to the logistics of running surgical RCTs as well as the competing priorities often faced by the affected patient population (ie, young male athletes) that may preclude their involvement from trials that limit their playing time. While several trials have sought to compare operative versus nonoperative management of FTSD, the statistical robustness of their findings is limited, with mean and median fragility indices of 3.5 and 2, respectively.^1,9^ As per the seminal study on the fragility index by Walsh et al,^51^ the median fragility index of RCTs assessing operative versus nonoperative management of FTSD falls below the 25th percentile of 399 trials published in high-impact journals. Further, the generalizability of RCTs assessing surgical management of FTSD is limited to a narrow patient population often studied by these trials, that of young, predominantly male patients. While epidemiologic studies indicate that young, male patients are at higher risk of shoulder dislocation, even when standardizing for type of sporting activity, this does not preclude focused studies on less represented patient populations.^49^ In terms of running larger trials to increase the statistical robustness of findings, several practical limitations exist such as a patient’s in-season status. As demonstrated by Buss et al,^8^ 90% of athletes with an in-season shoulder dislocation were able to return to sports at a mean time of 10 days after injury, with recurrent instability being noted in 37% of patients, an informed risk an athlete may wish to take to avoid missing an entire season for surgery.

### Limitations

This study is not without its limitations. First, to increase the number of included studies, this review included both RCTs and observational studies rather than limiting the inclusion to RCTs only. Nonetheless, meta-analyses stratified by study design (RCTs vs observational studies) demonstrated concordant results. Second, the included studies largely comprised young, male patients, which limits the generalizability of the findings. Third, the timing of surgery was heterogeneously reported across studies, ranging from as early as 1 day after dislocation to upward of 4 weeks. Further research is needed to evaluate the role of early intervention in this patient population. Fourth, follow-up periods varied notably across studies. However, the mean follow-up period for eligible studies was 54.2 months, which was sufficient in duration to adequately capture repeat dislocation or instability events.^56^ Included studies also lacked consistency in the reporting of recurrent instability events despite subjective instability, subluxation, and redislocation all having their unique adverse sequalae. To account for the more severe adverse outcomes associated with redislocation (ie, bone loss), this meta-analysis included all recurrent instability, subluxation, or redislocation events under cumulative instability rates while also presenting redislocation rates as a separate entity, where possible. Last, this meta-analysis solely compared ABR with nonoperative management of FTSD, whereas other surgical techniques do exist to manage the same or more complex sequalae of shoulder dislocation including open Bankart repair and the role of remplissage, as well as various bone restorative techniques including the Latarjet procedure. Future directions include both high-quality RCTs assessing the management of FTSD using modern surgical techniques in young male patients, as they are typically more affected by this pathology, and smaller case series that include less often affected patient populations such as female athletes.

### Summary of Recommendations

1. ABR in the setting of a first-time anterior shoulder dislocation is recommended in patients with known risk factors for recurrence including male sex, young age, and participation in a contact sport.
2. The timing of surgical management is multifactorial and should consider patient preferences including return to play timelines and/or employment obligations while also minimizing the risk of recurrence.
3. While advanced imaging is the gold standard for the assessment of bone loss, radiography should be effectively used to maximize diagnostic accuracy prior to consideration of CT and/or MRI, especially in single-payer health care systems. Recommended views include anteroposterior, lateral, and axillary views.
4. CT assessments of the shoulder are only recommended if there is concern for bone loss or bony Bankart fracture that may warrant bony reconstruction surgery.
5. MRI is of value particularly to assess for potential concomitant pathology (ie, rotator cuff tears or humeral avulsions of the glenohumeral ligament), and as such, timely access to MRI is critical to avoid delays in surgical care in the setting of FTSDs.

## Conclusion

Early ABR of first-time anterior shoulder dislocation consistently demonstrated decreased subsequent rates of cumulative instability events, shoulder redislocation, and revision surgeries, as well as improved patient-reported outcomes when compared with nonoperative management.

## Footnotes

¶¶ References 5, 7, 10, 11, 15, 28, 31, 35, 41, 44, 55.

## References 5, 10, 11, 28, 31, 35, 41, 44, 55.

## Appendix

**Table A1:** Search Strategy: MEDLINE, EMBASE, and CENTRAL via OVID Search Engine

1. exp Shoulder/ or shoulder.mp. 2. glenohumeral.mp. 3. exp Shoulder Joint/ 4. dislocation.mp. or exp Shoulder Dislocation/ 5. instability.mp. 6. stabilisation.mp. 7. stabilization.mp. 8. exp Bankart Lesions/ or bankart.mp. 9. latarjet.mp. 10. coracoid transfer.mp. 11. distal tibia.mp. 12. Surgery.mp. 13. Surgical.mp. 14. capsular shift.mp. 15. rotator interval closure.mp. 16. capsulorrhaphy.mp. 17. 1 or 2 or 3 18. 4 or 5 19. 6 or 7 or 8 or 9 or 10 or 11 or 12 or 13 or 14 or 15 or 16 20. 17 and 18 and 19 21. first time.mp. 22. traumatic.mp. 23. initial.mp. 24. acute.mp. 25. 21 or 22 or 23 or 24 26. 20 and 25
